# Molecular Mechanisms of 5-Fluorocytosine Resistance in Yeasts and Filamentous Fungi

**DOI:** 10.3390/jof7110909

**Published:** 2021-10-27

**Authors:** Fatima Zohra Delma, Abdullah M. S. Al-Hatmi, Roger J. M. Brüggemann, Willem J. G. Melchers, Sybren de Hoog, Paul E. Verweij, Jochem B. Buil

**Affiliations:** 1 Department of Medical Microbiology, Radboud University Medical Centre, 6252 AG Nijmegen, The Netherlands; FatimaZohra.Delma@radboudumc.nl (F.Z.D.); Willem.Melchers@radboudumc.nl (W.J.G.M.); paul.verweij@radboudumc.nl (P.E.V.); 2 Natural & Medical Sciences Research Center, University of Nizwa, Nizwa 616, Oman; abdullaalhatmi@gmail.com; 3 Centre of Expertise in Mycology Radboudumc/CWZ, Radboudumc Center for Infectious Diseases (RCI), 6252 AG Nijmegen, The Netherlands; roger.bruggemann@radboudumc.nl (R.J.M.B.); sybren.dehoog@radboudumc.nl (S.d.H.); 4 Foundation Atlas of Clinical Fungi, 1214 GP Hilversum, The Netherlands; 5 Department of Pharmacy, Radboud University Medical Center, 6252 AG Nijmegen, The Netherlands

**Keywords:** 5-fluorocytosine, resistance, molecular mechanisms

## Abstract

Effective management and treatment of fungal diseases is hampered by poor diagnosis, limited options for antifungal therapy, and the emergence of antifungal drug resistance. An understanding of molecular mechanisms contributing to resistance is essential to optimize the efficacy of currently available antifungals. In this perspective, one of the oldest antifungals, 5-fluorocytosine (5-FC), has been the focus of recent studies applying advanced genomic and transcriptomic techniques to decipher the order of events at the molecular level that lead to resistance. These studies have highlighted the complexity of resistance and provided new insights that are reviewed in the present paper.

## 1. Introduction

Significant advances in antifungal therapy have been achieved during the last few decades with the introduction of new generations of antifungal agents, improved fungal diagnostics, and standardization of in vitro susceptibility testing. Nevertheless, management and appropriate treatment of fungal infections remains a challenge that is aggravated by the emergence of antifungal resistance [1,2]. Antifungal drug resistance is associated with increased probability of treatment failure and mortality [3]. Elucidation of the molecular mechanisms of antifungal resistance may help to develop better diagnostic approaches and new therapeutic strategies that can overcome resistance and optimize the treatment efficacity.

5-Fluorocytosine (5-FC) is one of the earliest antifungal agents, initially synthesized in 1957 as an anticancer drug. Unlike 5-fluorouracil (5-FU), a closely related fluorinated pyrimidine, 5-FC does not exhibit antineoplastic activity, but was found to possess antifungal activity. The compound has been used since 1968 to treat human cryptococcosis and candidiasis [4]. 5-FC is active against ascomycete and basidiomycete yeasts, and some hyaline and melanized filamentous fungi [4]. The drug is particularly significant for treatment of fungal infections at body sites with limited drug penetration of other antifungal agents, such as infections of the urinary tract, brain and eyes, or heart valves [5]. Effective therapy of fungal infections of these sanctuary sites is challenging, as other drug classes, such as the lipophilic triazoles, echinocandins, and polyenes, show very limited penetration. The use of 5-FC as a monotherapy, however, is restricted by the rapid emergence of resistance, and is only recommended for some cases of chromoblastomycosis and uncomplicated lower urinary tract infection due to *Candida* species that are resistant to fluconazole [6]. When administered in combination with amphotericin B, 5-FC has become the gold standard for treating cryptococcal meningitis and complex *Candida* infections [7,8]. Although resistance to 5-FC is well known, the genetic basis of resistance regulation has remained enigmatic. An understanding of these mechanisms may help to discover novel ways to reduce or reverse resistance and broaden the clinical use of this drug, benefiting from its excellent tissue distribution. In recent years, several studies have revisited this classical drug to elucidate the molecular mechanisms of resistance in different fungi. In the present review, we discuss the mode of action of 5-FC and give an overview of the current resistance mechanisms that have been reported in the literature.

## 2. Spectrum of Activity

5-FC exhibits a broad spectrum of activity against common pathogenic yeasts. For *Candida* species, using the standardized National Committee for Clinical Laboratory Standards (NCCLS) antifungal susceptibility testing method, the MIC_90_ ranges from 0.12 to 1 µg/mL and the susceptibility rate is 95 to 100% for the following species: *C. dubliniensis*, *C. glabrata*, *C. kefyr*, *C. lusitaniae*, *C. glabrata*, and *C. albicans*. However, it is less effective against *C. krusei* (MIC_90_ 32 µg/mL and 5% sensitive/susceptible) [9]. 5-FC demonstrates in vitro activity against *Cryptococcus* species; the MIC values, determined according to the recommendations proposed by the European Committee for Antibiotic Susceptibility Testing of fermentative yeasts (AFST-EUCAST, definitive document 7.1), are higher for *C. neoformans* isolates than for *C. gattii* (MIC_50_ of 4 and 1 µg/mL, respectively) [10]. 5-FC is also active in vitro against some uncommon pathogenic fungi, including species of *Cyberlindnera*, *Debaryomyces*, *Diutina*, *Rhodotorula*, and *Saccharomyces,* while other yeasts exhibit systematically elevated MICs, for example *Cutaneotrichosporon mucoides*, *Dipodascus geotrichum*, *Trichosporon asahii*, *Yarrowia lipolytica*, and species of *Naganishia* and *Pichia* [11]. *Aspergillus* and some agents of chromoblastomycosis in vitro are less susceptible to 5-FC [4].

## 3. Indication

5-FC is clinically licensed for the treatment of cryptococcosis, chromoblastomycosis, and aspergillosis. 5-FC is a first-line therapy for the treatment of cryptococcal meningitis. It is administered in combination with amphotericin B as induction therapy [7]. Adding 5-FC to amphotericin B resulted in faster sterilization of the cerebrospinal fluid (CSF) [12].

This latter effect is also obtained with the combination of 5-FC with fluconazole as compared with fluconazole monotherapy. Furthermore, the association of 5-FC with fluconazole has shown a noninferiority with two weeks of amphotericin B-based therapy [13]. Thus, is also recommended in areas where amphotericin B is not available [14]. It has the advantages of being well tolerated, administered orally, and prevented treatment failure as it suppresses the amplification of hetero-resistance to fluconazole [15].

5-FC is also used as adjunctive with amphotericin B or fluconazole in treating complex systemic fungal infections, including septicemia, endocarditis, meningitis, or pulmonary infections caused by susceptible strains of *Candida* or *Cryptococcus* [6,8]. 5-FC monotherapy is limited only for the treatment of *Candida* cystitis in cases of fluconazole resistance given the high urinary concentrations of 5-FC and treatment of chromoblastomycosis caused by various melanized fungi [4,6].

## 4. Mode of Action

**Figure 1:**
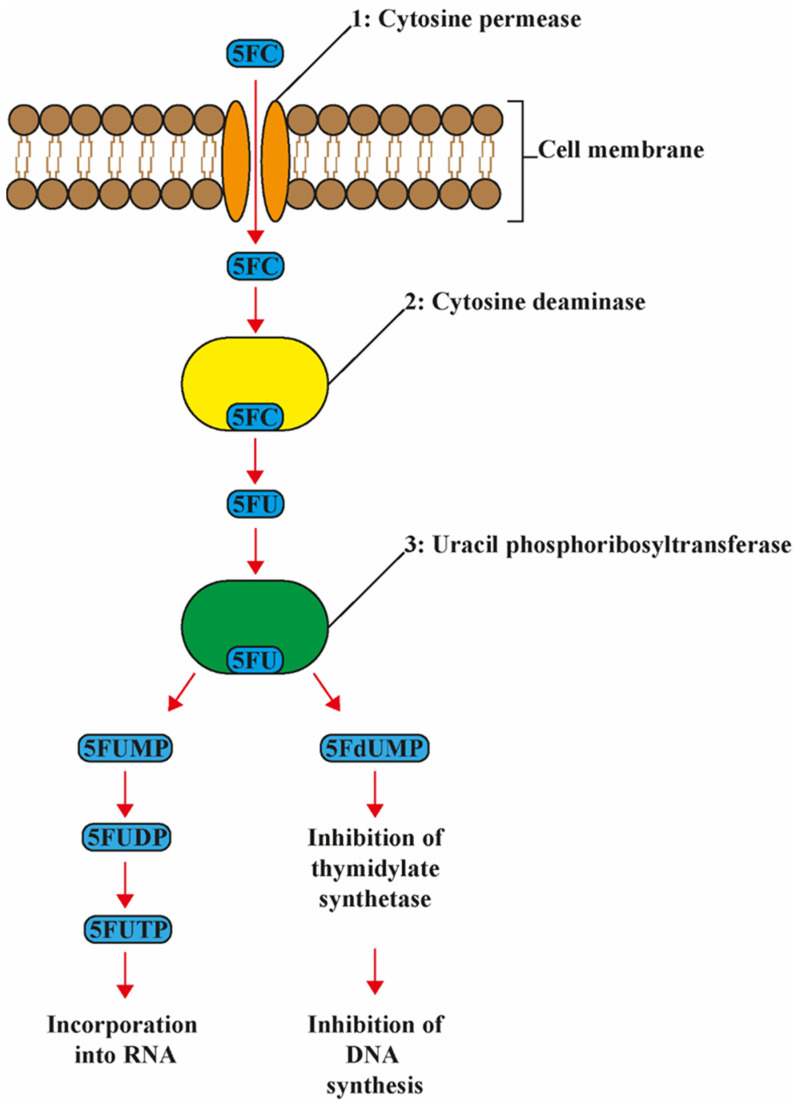
Schematic and simplified presentation of the mode of action of 5-flucytosine. 5-Flucytosine (5-FC) is transported into the cell by the enzyme 1: cytosine permease. Here 5-FC is deaminated to 5-fluoraucil (5-FU) by the enzyme 2: cytosine deaminase. Then, 5FU is converted to 5-fluorouridine monophosphate (FUMP) by the enzyme 3: uridine monophosphate pyrophosphorylase, then into 5-fluorouridine diphosphate (FUDP) and eventually into 5-fluorouridine triphosphate (FUTP). FUTP is incorporated into RNA altering the aminoacylation of tRNA and inhibiting protein synthesis. Furthermore, 5FU is converted to 5-fluorodeoxyuridine monophosphate (FdUMP) by the enzyme 3: uridine monophosphate pyrophosphorylase. FdUMP inhibits DNA biosynthesis via inhibition of thymidylate and incorporation into DNA.

The mode of action of 5-FC is unique among antifungal agents, as it targets DNA, RNA, and protein synthesis. 5-FC is a prodrug, i.e., for its activation the compound needs to be metabolized via the pyrimidine salvage pathway, where it acts as a subversive substrate with the subsequent production of toxic nucleotides and disruption of DNA and protein synthesis (Figure 1). After being actively transported into the fungal cell by membrane permeases (cytosine permease, encoded by the *FCY2* gene and other homologs encoded by *FCY21*, *FCY22*), 5-FC is converted via 5-FU to 5-fluoro-uridylate (synonymous with 5-fluoro-UMP (5-FUMP)) under the action of the enzymes cytosine deaminase, encoded by *FCY1*, and uracil phosphoribosyltransferase (*UPRT*), which is encoded by the *FUR1* gene, respectively. The 5-FUMP is in turn phosphorylated by two specific kinases to 5-fluoro-UTP, which is incorporated into the RNA. 5-FUMP is also reduced to 5-fluoro-2′-deoxyuridylate, which inhibits the enzyme thymidylate synthetase and thus DNA synthesis by decreasing the available nucleotide pool. Mammalian cells lack the enzyme cytosine deaminase. Therefore, 5-FC is not converted to 5-FU and consequently these cells are not directly subject to the toxic effects of 5-FC [3].

## 5. Pharmacokinetics

5-FC is available as an oral capsule and as an intravenous formulation [16], but is only available in a limited number of countries [17]. 5-FC is generally administrated at 100 mg/kg/day in 2–4 divided dosages. The oral bioavailability is approximately 75–90% in healthy patients [18,19]. A lower oral bioavailability of approximately 45% was found in late-stage HIV-infected patients from Thailand [16]. It is eliminated by the kidneys and either dose or frequency or both should be reduced in patients with impaired renal function [20,21,22]. 5-FC distributes well into various body sites and compartments. In a cohort of patients treated for cryptococcal meningitis, CSF 5-FC concentrations were 84% of the corresponding plasma concentrations [16].

## 6. Toxicity

5-FC is associated with gastrointestinal side effects (in 6% of patients treated with 5-FC) such as nausea, diarrhea, vomiting, and diffuse abdominal pain, but also more severe side effects such as hepatotoxicity and bone-marrow depression [23]. 5-FC exposure is limited by toxicity and therapeutic drug monitoring is needed to avoid toxic concentrations. 5-FC serum concentrations of 100 mg/L and above are associated with increased incidence of bone marrow depression and hepatotoxicity [23,24,25,26]. Furthermore, higher concentrations of 5-FU are detectable in plasma from patients that received oral 5-FC in comparison to intravenous 5-FC, possibly due to conversion of 5-FC to 5-FU by the intestinal microflora [16]. However, no differences in toxicity or treatment efficacy were observed [16]. Flucytosine is contraindicated in pregnancy [27].

## 7. 5-FC Resistance

The clinical use of 5-FC is limited by the common occurrence of primary (in subpopulations of *C. albicans* and *C. dubliniensis*) and acquired resistance, particularly when used as a monotherapy. Most of the studies on resistance mechanisms have focused on *S. cerevisiae* and *C. albicans*, and the detection of mutations in important enzymes of the pyrimidine salvage pathways. Results of these studies were reviewed previously [5,28,29]. It was shown that 5-FC resistance may result from loss or mutation of any of the enzymes involved in the activation of cytosine permease, cytosine deaminase, or uracil phosphoribosyltransferase or from the increased production of pyrimidines (Table 1). However, the absence of mutations in these enzymes in many clinically 5-FC-resistant isolates led to hypotheses of the existence of other mechanisms that might regulate the response of fungal cells, other than the genes involved in the pyrimidine salvage pathways.

To date, no studies have evaluated the mutant prevention concentration of 5-FC. Furthermore, it is not known whether resistance developed more often in sanctuary sites, where drug levels may be lower due to reduced 5-FC penetration. While resistance to 5-FC is a single-step process, 5-FC resistance may develop rapidly regardless of the dose or the site of infection when used as a single agent.

### 7.1. Saccharomyces cerevisiae

Mutations in three key enzymes of the pyrimidine salvage pathways, i.e., *FCY1*, *FCY2*, and *FUR1*, have been shown to cause 5-FC resistance [45,46]. While disruption of cytosine deaminase is associated with a strong dose-independent resistance, disruption of cytosine permease leads to a low but dose-dependent level of toxicity indicating involvement of two other cytosine permeases, *FCY21* and *FCY22*. In addition, two other *FCY2* homologues, *TPN1* and *FUR4*, and one encoded by *yOR071c*, were found to contribute significantly to 5-FC toxicity, thus revealing alternative 5-FC entry routes via other cytosine adenine permease homologues [47]. Kern et al. [30] correlated phenotypic 5-FC resistance to a single point mutation, Arg134Ser in the *FUR1* gene.

Additional resistance mechanisms have been reported that include upregulation of pyrimidines in de novo synthesis and a loss of function or disturbance in uridine monophosphate pyrophosphorylase [7]. Zhang et al. [48] applied genome-wide yeast DNA microarray and identified 96 responsive genes, of which 57 showed over two-fold upregulation and 40 were more than two-fold downregulated. Most of the upregulated genes, involved in DNA repair, synthesis, and replication, represented the highest proportion of induced genes that corresponded with the mechanism of action of 5-FC. Among the upregulated genes were *UBC5* and *UBC14*, both involved in the ubiquitin-dependent pathway, which is known to be related to DNA repair and selective removal of damaged or obsolete proteins. *UBC5* and *UBC14* are thought to be responsive to the faulty protein synthesis caused by 5-FC. The observed cellular resistance to 5-FC might also be enhanced by increased DNA repair in the cell. Among the downregulated genes, the pleiotropic drug resistance (*PDR*) genes may play a significant role. *PDR15*, which encodes a multidrug transporter protein, was downregulated by a factor of 2.3, indicating that 5-FC resistance may not be ascribed to extrusion by drug pumps in the cell membrane. Two enzyme-encoding genes, *CTS1* and *EGT2*, which function in the release of daughter cells from their mother cells, were downregulated by a factor of 3.7 and 10.2, respectively, indicating that 5-FC may also inhibit the separation of fungal cells. Other genes with significant repression were five genes related to protein synthesis; eight *ADE* genes that encode the enzymes of the purine nucleotide biosynthetic pathway; *INO1*, related to phospholipid synthesis; three genes, *PHO3*, *PHO5*, and *PHO11*, that contribute to increased phosphate levels; and the genes *ARE2* and *FAS2*, related to lipid metabolism.

### 7.2. Candida

Primary 5-FC resistance occurs in <5% of all Candida species, except for *C. krusei*, where resistance is detected in up to 35% of isolates [9,49]. Many factors have been shown to influence the development of the resistance in *Candida*, which varies with species, clade, or genotype, and also with ploidy and the presence of a phenotype mutator.

#### 7.2.1. *Candida albicans*

5-FC resistance in *C. albicans* has been reported to vary from 0 to 3% [50]. A relationship between 5-FC resistance and a decreased susceptibility to azoles was reported by Cuena-Estrella et al. [49]. Dodgson et al. [31] showed that both decreased susceptibility and increased resistance mostly correlate with a single change from cytosine to thymine at position 301 of the *FUR1* gene. This change results in an amino acid substitution from arginine to cysteine at position 101 in the *FUR1* protein. Strains homozygous (C/C) at position 301 were susceptible to 5-FC, whereas heterozygous counterparts (C/T) were less susceptible, and (T/T) homozygotes were fully 5-FC resistant. This mutation was restricted to Clade I strains. Hope et al. [32] identified the same mutation and found that the substitution C101R disturbs the quaternary structure of the enzyme, which was postulated to compromise its optimal activity. Additionally, another homozygous polymorphism in *FCA1* was described in this study that resulted in a glycine to aspartate substitution at position 28 in the cytosine deaminase.

Resistance was more frequent in *C. albicans* serotype B than in serotype A [51,52,53]. However, since the two serotypes have been shown to be interconvertible within a single strain [54], the association with 5-FC resistance that was found did not necessarily have a genomic background. Using rRNA biotyping, Mercure et al. [55] demonstrated that DNA type 1A (3.7 kbp) was mostly resistant to 5-FC, while DNA type IB comprised almost exclusively susceptible strains. Using DNA fingerprinting with the complex probe Ca3, Pujol et al. [56] demonstrated that all naturally resistant isolates of *C. albicans* were members of group 1, and 72% of the isolates of this group had 5-FC MICs >0.5 µg/mL, compared to 2% of non-group 1 isolates.

#### 7.2.2. *Candida dubliniensis*

*C. dubliniensis* was first described in 1995 as a sibling of the predominant human pathogen *C. albicans*. Three well-defined intraspecific clades can be distinguished, the species being significantly less diverse than *C. albicans* [57]. Resistance to 5-FC in *C. dubliniensis* is exclusively found in isolates of Clade 3 and was correlated to a homozygous S29L mutation in the cytosine deaminase gene [33,34,35]. McManus et al. [35] proved, using genetic transformation methods, that this mutation is responsible for the resistance to this drug. This radical substitution results in the replacement of a hydrophilic polar amino acid (serine) with a hydrophobic nonpolar residue (leucine) in the β1 strand of the cytosine deaminase enzyme and is closely linked to an active-site residue. The substitution may lead to disruption of the quaternary structure of the enzyme, distorting the active site and inhibiting the conversion of the 5-FC prodrug to its toxic form, 5-FU [35].

#### 7.2.3. *Candida lusitaniae*

*C. lusitaniae* has a remarkable ability to develop antifungal resistance, mainly to amphotericin B but also to azoles, echinocandins, and 5-FC. Many studies have aimed to decipher the molecular mechanisms of 5-FC resistance and its cross resistance with fluconazole. It was shown that inactivation of the genes *FCY2*, *FCY1*, and *FUR1* in *C. lusitaniae* produced two patterns of 5-FC resistance. The mutant *FUR1* also demonstrated resistance to 5-FU, whereas mutants *FCY1* and *FCY2* demonstrated fluconazole resistance in the presence of subinhibitory 5-FC concentrations, and extracellular 5-FC would behave as a competitive inhibitor of fluconazole uptake [58,59]. Florent et al. [36] demonstrated, by using yeast transformations, that two genetic events are responsible for both 5-FC and 5-FC/fluconazole resistance in clinical isolates of *C. lusitaniae*: either the nonsense mutation C505T in *FCY2* and its putative nonfunctional truncated purine cytosine permease of 168 amino acids unable to transport 5-FC, or the missense mutation T26C in the *FCY1* gene, result in an amino acid replacement M9T in cytosine deaminase.

Kannan et al. [37] used comparative genomics to elucidate the mechanism of multidrug resistance in *C. lusitaniae* and described a new mechanism of 5-FC resistance with a mutation in a putative transcriptional activator (MRR1). The V668G substitution was responsible for the upregulation of the MFS7, a multidrug transporter that mediated the resistance to 5-FC and 5-FC/fluconazole cross-resistance.

#### 7.2.4. *Candida glabrata*

*C. glabrata* has emerged in recent years as the second most common agent of mucosal and invasive candidiasis [60]. The yeast is characterized by a high frequency of acquired resistance to multiple classes of antifungals drugs. This may be related to the particular genetic background of this species, as no sexual reproduction is known. In contrast to *C. albicans*, *C. glabrata* possesses a haploid genome and therefore a single mutation in an appropriate gene may result in acquired antifungal resistance phenotype. Recently, genetic defects in the mismatch repair (MMR), the DNA repair pathway due to the presence of *MSH2* mutations, were described to enhance the development of resistance [61]. Contrary to expectations, resistance of *C. glabrata* to 5-FC is reported to be 1%, with resistant mutants occurring at a relatively low frequency of 2 × 10^−7^ [9]. Normak and Schonebek [62] analyzed pyrimidine incorporation and *UPRT* activity of five *C. glabrata* clinical isolates and demonstrated a decreased *UPRT* activity in one of them. Fasoli et al. [63] investigated the metabolism of fluoropyrimidines in a selected mutant of *C. glabrata* by nuclear magnetic resonance, which revealed a partial loss of cytosine permease activity. Chapeland-Leclerc et al. [38] provided indirect evidence without formal proof that a defective *FUR1* protein bearing a G190D substitution was responsible for the fluoropyrimidine resistant phenotype, suggesting that the mechanism of resistance to 5-FC is due to mutations that result in enzyme deficiency. Multiple mutations were also described in in vitro 5-FC resistant mutants of *C. glabrata* in the main enzymes of the pyrimidine salvage pathway. Mutations in *FUR1*, i.e., I83K and D193G, were responsible for the replacement of charged with uncharged residues. The ΔG73-V81 mutation involving a 9-amino-acid deletion was found in isolates with 5-FC/5-FU cross-resistance, while strains with mutations in *FCY1* (A15D, G11D, and W148R), and *FCY2* (G246S and I384F) were not cross-resistant to 5-FU [39,40]. Edlinde et al. [39] confirmed, by isolating the mutants in URA3 strains and then testing their ability to assimilate the cytosine, that the mutants are null. They also found some of these mutations (*FUR1* G210D and L136R, *FCY1* T84L, and *FCY2J* I384F) in clinical isolates. 5-FC-azole antagonism was described in *C. glabrata* as a result of upregulation of the Pdr1-dependent CDR1 induced by 5-FC [64].

#### 7.2.5. *Candida tropicalis*

In a study by Cuenca-Estrella et al. [49], 5-FC showed potent in vitro activity against all 117 tested isolates of *C. tropicalis*. However, Desnos-Olivier et al. [41] found 5-FC resistance in 45 of 130 (35%) isolates and suggested that resistance to 5-FC in *C. tropicalis* could be clonal and represent a subgroup of *C. tropicalis*. The 5-FC resistant isolates did not carry any mutation in the coding sequences of *FCY1*, *FCY2*, or *FUR1*, but a K177E mutation was suggested without formal proof in the *URA3* gene. The *URA3* enzyme (orotidine 5′-phosphate decarboxylase, ODCase) is involved in the metabolic pathway of uridyl-monophosphate (UMP), which is a substrate of thymidylate synthetase and UMP kinase, both involved in nucleic acid synthesis. The ODCase is not known to interfere directly with 5-FC activity. However, one of the resistance mechanisms against 5-FC concerns increased transcription of all genes involved in the de novo pyrimidine biosynthetic pathway (including *URA3*) leading to an overabundance of UMP [41].

Chen et al. [42] found that 22 out of 30 5-FC susceptible isolates with resistant progeny after exposure to the drug had a heterozygous G/T at the 145^th^ position of *FCY2* encoding purine–cytosine permease, whereas their progeny recovered from within the inhibitory ellipses had homozygous T/T. They proved, using transformation approaches, that this mutation resulted in null alleles for both copies of the gene and produced only truncated proteins, leading to 5-FC resistance. The authors concluded that *C. tropicalis* strains containing heterozygous alleles may develop 5-FC resistance readily, whereas patients carrying strains with homozygous G/G wild-type alleles can be treated with 5-FC.

#### 7.2.6. *Candida auris*

Since its first description in 2009, *C. auris* has emerged as a multidrug resistant pathogen. It is associated with high mortality and therapeutic failures because of its ability of colonization and its resistance to a broad spectrum of antifungals, especially to fluconazole and amphotericin B [65].

5FC is active against *C. auris*; the rate of resistance varies from 0 to 47% [66]. The molecular mechanisms of resistance to 5FC in this pathogen are still unknown. Rhodes et al. [43] reported in one 5-FC resistant isolate a mutation in the *FUR1* gene that causes an amino acid substitution from isoleucine to phenylalanine at position 211. Bhattacharya et al. [67] demonstrated that transient gene duplication can occur in *C. auris* during replicative aging, responsible for tolerance to 5-FC and other antifungal drugs in older *C. auris* cells (10 generations) compared to younger (0–3 generations cells).

### 7.3. Cryptococcus

The molecular mechanism of 5-FC resistance of *C. neoformans* is not well understood but has been suggested to be related to mutation in pyrimidine salvage enzymes, similar to *C. albicans* [28], despite large phylogenetic distances between asco- and basidiomycetous yeasts. First studies indicated that 5-FC resistance was not drug-induced, was stable, and was nearly always accompanied by 5-FU resistance. 5-FC mutation rates ranged from 1.2 to 4.8 × 10^−7^ per cell division [68]. Whelan et al. [28] ascribed this resistance to a reduction in *FUR1* activity, based on a high frequency of cross-resistance to 5-FU. In *C. gattii*, deletions of *FCY2* conferred resistance to 5-FC [69]. However, recent studies of 5-FC resistant *C. gattii* isolates presented no mutations in *FCY1*, *FUR1*, or any of three putative *FCY2* paralogs that should explain drug resistance [70]. Using comparative transcriptomics, Song et al. [71] identified 177 responsive genes to 5-FC in *C. neoformans*. Among these, an APES-like transcription factor *Mbs1* was present, which has pleiotropic functions in *C. neoformans*, including regulation of susceptibility to diverse antifungal drugs such as 5-FC, azoles, and polyene drugs. The gene further played a role in ergosterol biosynthesis, cell membrane stability, genotoxic, oxidative, and salt stress responses, in vitro and in vivo morphological differentiation, and virulence. Jung et al. [72] showed that 27 transcription factors (TFs) differentially regulate 5-FC resistance and described some TF mutants with increased resistance (*HLH3, RIM101, GAT204, HOB3, FZC50, ZNF2, RDS2, FZC31*) as well as TF mutants with increased susceptibility (*NRG1*, *ZFC2*, *YAP1*, *MBS1*, *FZC6*, *YAP2*, *BZP3*, *JJJ1*, *HLH1*, *PIP2*, *APN2*, *FZC46*, *HAP2*, *FZC51*, *BZP5*, *HCM1*, *FZC19*, *BZP2*, *FZC44*).

Billmyre et al. [44] showed that resistance to 5-FC in *C. deuterogattii* is acquired more frequently, greater than 15-fold in *msh2Δ* mutants with defects in DNA mismatch repair, which consequently confers an elevated mutation rate. All 29 resistant isolates used in this study were 5-FC/5-FU resistant. It was expected as previously described that these isolates harbor mutations in *FUR1*. However, this was confirmed in only 12 isolates; the *FUR1* mutations occurred through multiple mechanisms, including regional deletions, homopolymer tract length changes that introduced frameshift mutations, and a splice site acceptor point mutation. Furthermore, the authors described a new mechanism of resistance, i.e., a mutation in the UDP-glucuronate decarboxylase 1 (*UXS1*) gene may confer resistance to both 5-FC and 5-FU. The *UXS1* gene encodes the enzyme that converts UDP-glucuronic acid to UDP-xylose. This pathway is critical for the formation of the capsule, a core virulence trait of *Cryptococcus*, and for synthesis of other glycoconjugates. In this study, four loss of function mutations in *UXS1* were identified: a single base deletion in a 3xT homopolymer, a single base insertion in a 7xC homopolymer, and two missense mutations, Y217C and D306G. The latter mutation is identified in the only isolate with *FCY2* mutation (W167Stop). Mutations in *UXS1* lead to the accumulation of UDP-glucuronic acid and alterations in the nucleotide metabolism, which appear to suppress the toxicity of both 5-FC and its toxic derivative 5-FU. The role of the mutation in *UXS1* and some regional deletion including *FUR1*, which are associated with loss of virulence, is still unclear in the resistance to 5-FC in patients. Additionally, the authors found no mutation in *FCY1*, *FCY2*, *FUR1*, or *UXS1*, in eight remaining isolates that could explain the resistance to 5-FC; the existence of other unknown mechanisms may be presumed [44].

### 7.4. Aspergillus

5-FC is rarely used in the treatment of *Aspergillus* diseases, due to an apparent lack of activity of 5-FC in vitro. The compound is not recommended for therapy of any form of aspergillosis by the Infectious Diseases Society of America [73]. However, it would be more accurate to state that 5-FC has limited activity against most *Aspergillus* isolates in vitro, with MICs ranging from 0.25 to >256 µg/mL using NCCLS [74]. This contradicts evidence showing that 5-FC treatment is able to significantly improve survival in a murine model of infection [75,76]. One explanation for the lack of in vitro/in vivo concordance is the variable activity of 5-FC at pH 5 versus pH 7. Lower pH has been shown to increase 5-FC activity against *A. fumigatus* up to 4000-fold [77]. MICs determined at low pH better reflect results of in vitro models of infection for *A. fumigatus*. At neutral pH, two transcriptional regulators, PacC and the CCAAT binding complex (CBC), orchestrate 5-FC resistance via negative regulation of the gene encoding, the purine cytosine transporter, and *FCY2* orthologue FCYB [77]. The authors also demonstrated that *FCYB* is critical for 5-FC activity, and the reduced expression of *FCYB* at pH 7 is the major mechanism conferring intrinsic 5-FC resistance in *A. fumigatus* [77]. However, Birštonas et al. [78] found that the ΔfcyB mutant strain was not able to grow at pH 5 and showed severe growth inhibition at pH 7, which indicated the existence of other uptake mechanisms. In contrast to the ΔfcyB mutant, the ΔfcyA and Δuprt mutants displayed full resistance to 5-FC up to 100 μg/mL, regardless of the pH. Furthermore, 100 μg/mL of 5-FU blocked growth of wt, ΔfcyB, and ΔfcyA strains at pH 5 as well as pH 7, while the Δuprt mutant was resistant. Furthermore, the inactivation of these genes caused no adverse effects with respect to nutrient requirements, stress resistance, or virulence in *A. fumigatus*, which make these genes a potential novel genetic toolbox for targeted genomic insertions of DNAs of interest (DOIs) in the genetic engineering of fungal species.

### 7.5. Dermatophytes

5-FC has no activity against dermatophytes; high MIC values were previously described [79,80]. Zhao et al. [81] performed cDNA microarray analysis to determine the response of *Trichophyton rubrum* to 5-FC and found that 474 genes were differentially expressed, of which 196 showed increased and 278 decreased expressions. Marked downregulation of genes involved in nucleotide metabolism (such as CDC21), transcription (such as E2F1), and RNA processing (such as SGN1, RIM4, and NOP1) was observed. Other genes which could be also affected by 5-FC have functions in signal transduction, chaperones, inorganic ion transport, secondary metabolite biosynthesis, amino acid transport, and lipid transport.

## 8. Conclusions

Although 5-FC resistance has been a well-known issue for a long time, the literature review indicates that our current knowledge regarding molecular mechanisms controlling 5-FC resistance is still limited. Resistance cannot be explained only by the alteration of the main target of this drug; it is more complex and implicates the involvement of different pathways depending on the fungal species. More work is needed, especially now that advanced molecular techniques have become available, allowing more in-depth exploration of the global response of fungi to antifungals. The use of these molecular techniques should be more oriented towards the analysis of resistant isolates that emerge in vivo in treated patients and can be compared with drug susceptible parent strains. This will allow all possible targets responsible for the development of 5-FC resistance to be identified. Furthermore, the availability of gene editing technology will provide important proof of the contribution of these mutations in 5-FC resistance. These studies will further increase our knowledge of the genetic basis of resistance to 5-FC and help to identify new potential diagnostic and treatment targets that could prevent the emergence of the resistance and may lead to a more appropriate use of this antifungal agent.

## Figures and Tables

**Table 1:** Mechanisms of resistance to 5-FC.

Species	Isolates	Gene	Gene Function	Mutation	Consequence	Reference
***S. cerivisae***	In vitro	*FUR1*	Uracil phosphoribosyltransferase	R134S	Resistance to 5FU	[30]
***C. albicans***	Clinical	*FUR1*	Uracil phosphoribosyltransferase	C301T ---> C101R	Disruption of dimerization of the enzyme	[31,32]
	Clinical	*FCA1*	Cytosine deaminase	G28D S29L	Defective enzyme	[32]
		*FCY2*	Cytosine permease	A176G	Defective enzyme	[32]
***C. dubliensis***	Clinical	Cd*FCA1*	Cytosine deaminase	C86T ---> S29L	Disruption of the quaternary structure of the enzyme	[33,34,35]
***C. lusitaniae***	Clinical	*FCY1*	Cytosine deaminase	T26C --->M9T	Defective enzyme	[36]
		*FCY2*	Cytosine permease	C505T--->E169Stop	Truncated cytosine permease	[36]
	Clinical	*MRR1*	Transcriptional activator	V668G	Upregulation of the multidrug transporter MFS7	[37]
***C. glabrata***	Clinical	*FUR1*	Uracil phosphoribosyltransferase	G190D	Defective Fur1p	[38]
	In vitro	*FUR1*	Uracil phosphoribosyltransferase	I83K, D193G	Defective enzyme	[39,40]
	In vitro	*FUR1*	Uracil phosphoribosyltransferase	ΔG73-V81	Defective enzyme	[39,40]
	In vitro	*FCY1*	Cytosine deaminase	A15D, G11Dand W148R	Defective enzyme	[39,40]
	In vitro	*FCY2*	Cytosine permease	G246S and I384F	Defective enzyme	[39,40]
		*FUR1* *FCY1* *FCY2J*	Cytosine deaminase	G210D L136R T84L I384F	Defective enzyme	[39,40]
***C. tropicalis***	Clinical	*URA3*	ODCase	K177E	Overabundance of UMP	[41]
		*FCY2*	Cytosine permease	G145T	Truncated protein	[42]
***C. auris***	Clinical	*FUR1*	Uracil phosphoribosyltransferase	F211I		[43]
***C. deuterogattii***	In vitro	*FUR1*	Uracil phosphoribosyltransferase	1134delT 1136delT 1440delA	Defective enzyme	[44]
		*FCY2*	Cytosine permease	W167Stop	Defective enzyme	
		*UXS1*	UDP-glucuronate decarboxylase 1	D306G Y217C 1520delT 1182insC	Accumulation of UDP glucuronic acid	

## Data Availability

Not applicable.
